# Supplementary figures and images for: Effects of Bacterial and Fungal Inocula on Biomass, Ecophysiology, and Uptake of Metals of Alyssoides utriculata (L.) Medik
Source: Plants (Basel). 2023 Jan 26;12(3):554. doi: 10.3390/plants12030554 (PMC9921704; doi:10.3390/plants12030554)

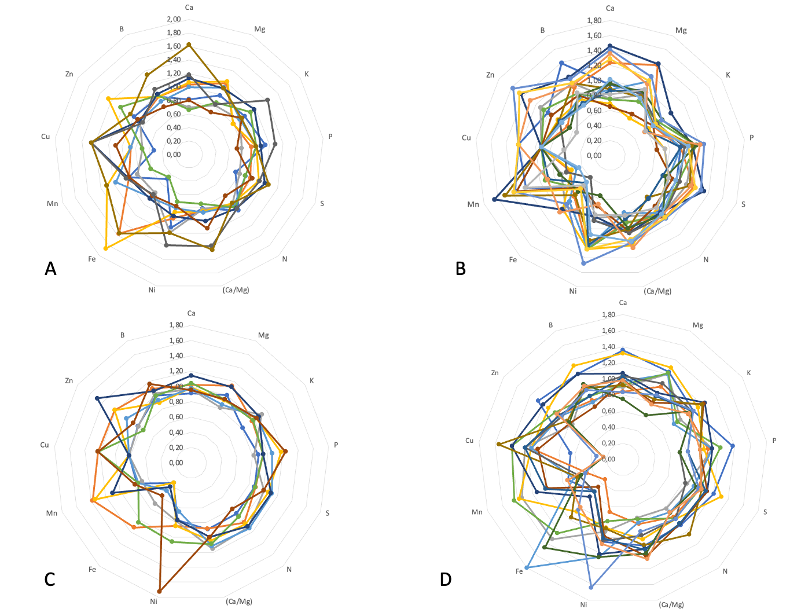

Supplement: Supplementary file 1 [file plants-12-00554-s001.zip › Figure S1.png]
